# Hazards inherent in interdisciplinary behavioral research

**DOI:** 10.1186/1742-9994-12-S1-S21

**Published:** 2015-08-24

**Authors:** David Crews, Seth A Weisberg, Sahotra Sarkar

**Affiliations:** 1 Department of Integrative Biology, The University of Texas at Austin, Austin, TX 78712, USA; 2 Institute of Neuroscience, The University of Texas at Austin, Austin, TX 78712, USA; 3 Departments of Integrative Biology and Philosophy, The University of Texas at Austin, Austin, TX 78712, USA

**Keywords:** transgenerational, synchronicity, ancestral, proximate, nature versus nurture, epigenetics

## Abstract

Many, if not all, questions in biology and psychology today were formulated and considered in depth, though typically in a different language, from the 1700's to the early 1900's. However, because of politics or fashion, some topics fell out of favor or failed to recruit new scientists and hence languished. Despite greatly expanded scholarship in the history of the life sciences in the twentieth century, many such topics have had to be rediscovered in recent years, while much of the wisdom already accrued stays in the older literature and not in active minds. This is particularly true today when scientific advances appear at breakneck speed. It would not be an exaggeration to say that many ‘breakthroughs’ turn out really to be rediscoveries of forgotten observations. Two areas of particular significance to the interdisciplinary study of behavior are the Norms of Reaction (from Biology) and the concept of Plasticity (from Psychology). These and related fields benefit from the perspective of epigenetics so long as rigorous operational definitions are implemented. It is also important to revive Hogben's admonition that the interaction of hereditary and environment cannot be understood outside of the context of development. Five examples of increasing complexity in phenotypic plasticity in brain and behavior are presented to illustrate this perspective.

## Introduction

There is an apparent need of each new generation of scientists to implement a new vocabulary to describe old concepts; in other words, to put old wine into new bottles or, for what will follow, new phonemes. Take, for example, the efforts to understand how the individual develops, a central question from the modern origins of both biology and psychology. Prior to the discovery of heritable units, this represented a debate that extends back in the Western intellectual tradition to Heraclites and Parminedes in ancient Greece. The alternate views of preformationism and epigenesis was settled in 1759 in the *Theoria Generationis* of Caspar Friedrich Wolff (1759) (see [1,2] for a thorough discussion of the history of these concepts during that period). Namely, that organisms develop in successive stages, increasing in complexity of organization, and not by the unveiling of ever larger yet fully formed individuals. It is curious these opposing views of nature re-emerged in the 19^th^century (e.g. [3]) and still can be found today in many arguments about the relative importance of nature and nurture in phenogenesis which have been shown to be vacuous several times during the twentieth century [4-11,5]. Despite the obviousness of the answer, there continues to be misunderstanding and confusion.

Modern discussions of the question of nature and nurture began with the work of Francis Galton in the 1860s (e.g. [12]). Relying on data from rudimentary twin studies, for most human traits, Galton opted for a defining role for nature, interpreted as what was contributed by heredity; this formed the basis for his lifelong advocacy of eugenics (a term he coined) for the improvement of the human race. Galton was also one of the founders of statistics and the progenitor of the “biometrical” or statistical study of populations that was furthered by his protégé, Karl Pearson (another major figure in statistics) along with W. F. R. Weldon [8,13]. It should be noted that then (as now), the seductive purity of mathematics and (later) classical genetics must be contrasted with the untidy nature of life (recognized, among others, by Galton's cousin, Darwin); this difference between theory and reality created some of the scientific schisms that persist today.

Although for a time epigenesis prevailed, August Weismann's germ plasm theory (i.e., hereditary specificity cannot pass from soma to germ plasm to alter what is transmitted through heredity in the next generation) [14], along with the then new field of genetics in the early twentieth century, led to the dominance of a genocentric program for biological research [8,15]. Along with R. A. Fisher's introduction of the analysis of variance (ANOVA), these three advances (classical genetics, germ plasm theory, ANOVA) contributed to many unintended and unnecessary controversies about the presumed separability of nature and nurture in development, with its more modern form being the partitioning the phenotypic variance into a heriable or genotypic (G) component, and environmental (E) component and a gene-environment (G x E) interaction component. Thus, the question, rephrased, became how the genotype results in the phenotype. Despite repeated demonstrations that the question is actually nature *and* nurture with genes *and* environment shaping phenotypes, the polarization of science was well underway.

If we assume that individual variation is the crux of all biological processes and, further, that uniformity leads to stasis while variation results in dynamic change, making evolution possible, then it is useful to trace in broad strokes the ideas that have been voiced over the past two centuries. This lays the foundation for recent studies in epigenetics. The thesis here is that we have long known the answer; we have just had difficulties (or are dissatisfied) with framing the question.

There are four sections to this paper. First, we will briefly summarize historical advances on this topic in biology, focusing mainly on the Norm(s) of Reaction (NoR) and in psychology, the concept of plasticity. In the second section we discuss the field of epigenetics, its origins and application today, establishing both a nomenclature for sorting out this increasingly complex literature and showing the multiple levels of interactions. In the third section we consider five examples of increasing complexity: (1) How incubation temperature during embryogenesis in the turtle acts on a common gene network to yield two distinct gonadal phenotypes, testes (male) and ovaries (female). (2) How incubation temperature during embryogenesis in the gecko results in intrasexual (vs intersexual) differences during adulthood and how both adult experience and age interact with embryonic temperature to shape the adult behavioral phenotype. We will then turn to mammalian systems and examine the relative contribution of important aspects of early life history. (3) We illustrate how the sex ratio of the litter in rats alters the brain and behavior when males attain adult status, and how sexually receptive females perceive these males. (4) The next example injects the complication of the nature of the individual's genotype with that of sex ratio of the litter in genetically modified mice. The results of this work shows how the established diagnostics of genotype can be completely masked by the sex and genotype of sibs in the litter. (5) Finally, we turn to the question of how inherited and acquired experiences combine to create new phenotypes. In the Fourth and final section we provide some guidance for future research in the field.

## Review

### A short history

There have been many attempts to develop principles that might govern individuality and its inheritance, in other words, how the genotype and environment interact to create the individual. This section will briefly summarize how this issue has historically been approached in both biology and psychology.

**A. Biology and the Norms of Reaction (NoR).** After the recovery of Mendel's work in the West (the exceptional case of Russia will be discussed below) around 1900, within biology the first significant attempt to understand the non-genetic factors that shaped the organismic development was through the use of the concept of NoR [16-18]. Woltereck introduced the NoR in the context of a study of morphological responses to environmental differences by different parthenogenetic pure lines of Daphnia and Hyalodaphnia species from German lakes [19]. He introduced “phenotype curves” that showed, for each genotype, the (quantitative) phenotypic response to environmental changes and defined the NoR as the totality of these curves for the complete set of genotypes. [In later work, starting in the 1920's, each of Woltereck's individual phenotype curve came to be called a NoR. We follow this usage in the rest of this paper.] For Daphnia and Hyalodaphnia, Woltereck found that various quantitative traits were affected by some environmental factors such as nutrient levels, and yet independent of others such as ambient temperature; further, these traits varied cyclically with other factors such as seasonality. For Woltereck, each of these curves represented a capacity that was inherited; he identified that capacity with what Johannsen [20] had called the “genotype,” an identification that was accepted as being essentially correct by [21]. Shortly afterwards, and apparently entirely independent of Woltereck and Johannsen, Nilsson-Ehle used “plasticity” to refer to a capacity of genotypes that produced different phenotypes in different environmental contexts of development [22].

NoRs represent the phenotypic values of genotypes as functions of environmental parameters and permit the visualization of differences in phenotypic response of different genotypes. NoRs are particularly easy to construct for many plants and other organisms that reproduce asexually since it makes it easy to create many individuals of identical genotypes. What NoRs demonstrate is the complexity of the developmental process that leads to phenotypes: how the same genotype reliably produces different phenotypes in different environments have generalized the NoR concept to groups of related genotypes [23].

Though Woltereck's experiments were widely discussed, especially in Germany (e.g. [24-26]), the NoR was rarely invoked in Western genetic literature until it was brought to general attention by Dobzhansky, an emigré from the Soviet Union to the United States (where he initially worked with Morgan in his Drosophila laboratory and eventually succeeded him at Columbia University) [27]. The reason seems to be the emergence of a genocentric ideology in Western genetics in the 1920's: that is, an assumption that genetic factors dominate phenogenesis [8]. William Bateson had defined “genetics” as a field of study as early as 1905; the work of the Morgan school, that is, the creation of a catalog of spontaneous mutations (starting with Morgan, [28,29]), and the determination of the rules of chromosomal mechanics established that these discrete heritable units, designated as “genes” by Johannsen [20], could be studied in a reliable and quantitative manner. The work of the Morgan school gave a material chromosomal basis for the formal (statistical) genetics of Mendel and his successors in the twentieth century.

When developmental complexity contradicted genetic expectations, for instance, in the case of the mutations analyzed by Romashoff that will be discussed below, new properties were attributed to genes so as to maintain genetic etiology: e.g., in 1926, a neuroanatomist Vogt [30] introduced the terms “penetrance” and “expressivity” to show how there could be a gene for a trait even when the presence of the gene did not lead to any manifestation of the trait (incomplete penetrance) or led to variable manifestations of the trait (variable expressivity) ([31] provide a history). Meanwhile, the 1920s saw the creation of a modern evolutionary theory based on the new genetics, adding to the prestige of the genetical point of view [13,32,33]; what also helped the entrenchment of genetics during this period were the many systematic refutations of the possible inheritance of acquired characters [34].

A related development that helped marginalize the NoR started with the development of analysis of variance by Fisher as mentioned earlier [35,36]. Fisher's [35] mathematical formalization of Mendelism, so as to show that Mendelian assumptions led to the earlier “biometrical” laws governing trait distributions, deconstructed the phenotypic variability using a linear model, a technique that came to be called the analysis of variance. Typically, in such a model there are three components, the genotypic and environmental components, and their interaction, namely G X E_B_; this “B” for “biometrical” is used to contrast Fisher's conception with that of Hogben (see below). Wright [37] reified the model to distinguish between three genotypic components of variability of a continuous trait: (i) additive effects of alleles at all loci; (ii) effects of dominance at each locus; and (iii) the result of interaction between loci (epistasis). This became the primary tool in Gene X Environment studies in both biology and psychology. Fisher assumed that correlation is a straightforward guide to causality that, even at that time, was recognized as inadequate. Eventually, the analysis of variance led to the introduction of the concept of heritability by Lush [38]; “broad” heritability is the ratio between the part of the phenotypic variance attributed to the genotype and the total variance; “narrow” heritability is that ratio using only the additive component of the genotypic variance. If these are high, it became customary to claim a genetic etiology (that is, the environmental contribution and the genotype-environment interactions were supposed to be unimportant). [There have been many critiques of the use of heritability and even Fisher [39] rejected it—for a summary of these arguments, [8].]

Use of NoRs for the analysis of phenogenesis was primarily developed within genetics in the erstwhile Soviet Union [16]. It is a common misconception that Mendel's work was completely ignored everywhere until the early 1900's. Mendel's findings were recognized and appreciated by the Russian botanist Ivan Fredorivich Schmalhausen (father of Ivan Ivanovich Schmalhausen) in Mendel's own lifetime [40]. The first experimental program that explicitly addressed the complexity of phenogenesis and phenotypic variability emerged in the Soviet Union in a research group formed around Chetverikov [41]. In 1922, Romashoff discovered the “abdomen abnormalis” mutation in *Drosophila funebris* that resulted in the degeneration of abdominal stripes. Individual variability in the mutant phenotype depended on environmental factors and Romaschoff [42] interpreted this dependence as a difference in the strength of the mutation's effect. This particular example was cited by Hogben as an example of the developmental relationship between genotype and environment [4]. [It also led to the introduction of the concepts of penetrance and expressivity, mentioned earlier, in a deliberate attempt to maintain a genetic etiology in contrast to Hogben [31,43].]

Meanwhile, for the “abdomen abnormalis” mutation, Dobzhansky pointed out that the mutant phenotype was not manifested for generations if the food was dry [27]. However, it reappeared if the offspring were supplied with moist food. Dobzhansky reasoned that this and other such examples showed that, even when environmental factors induced a trait, an unchanged NoR continued to be inherited according to Mendel's rules.

The Soviet school made a sharp distinction between adaptive and non-adaptive NoRs [16]. The former were incorporated into models of “organic selection,” originally proposed by Baldwin, Osborn, and Lloyd Morgan [44-46], but ignored subsequently, and then independently formulated in the Soviet Union by E. J. Lukin and others around 1936. The most influential version of this theory is found in I. I. Schmalhausen's *Factors of Evolution*[47]. His term for organic selection was “stabilizing selection.” Stabilization consisted of the replacement of an adaptive phenotypic response by an identical genotypic one, ensuring its transmission to future generations. Gause developed similar ideas [48-50].

Dobzhansky became the major proponent of the study of adaptive NoR and brought this focus with him when he moved from the Soviet Union to the United States in 1927 [51]. In his 1937 *Genetics and the Origin of Species*, Dobzhansky reintroduced the NoR to the Anglophone world: “one must constantly keep in mind the elementary consideration which is all too frequently lost sight of in the writings of some biologists; what is inherited in a living being is not this or that morphological character, but a definite norm of reaction to environmental stimuli. . . . [A] mutation changes the norm of reaction” (Pp. 169) [27]. Gathering data on adaptive norms was an important part of Dobzhansky's research program to elucidate the genetics of natural populations of various Drosophila species [52].

Since their introduction NoR have been systematically used to study plants [53] as well as several non-mammalian systems, including various Drosophila species [52,54,55], the spider mite *Tetranychusurticae*[56], the freshwater snail Physaheterostropha [57], and larvae of the wood frog *Ranasylvatica*[58,59]. Anticipating current debates on whether there are gene(s) for plasticity, Dobzhansky argued, following Woltereck, that what was inherited was not a trait but a NoR [60].

**B. Psychology and the Concept of Plasticity.** Psychology began with philosophers’ fascination with the human mind. Questions about the brain have an ancient history. Hippocrates of Cos, usually considered as the father of western medicine, wrote in the 5^th^ century BC that the brain was the seat of perception, sensation, and cognition and blamed diseases of emotion and sanity on malfunctions of this organ. Aristotle wrote about the necessity of repetition for memorizing facts, but believed cognition resided in the heart, with the brain acting merely as a radiator to cool the blood. He also originated the idea of the mind as a *tabula rasa*, a blank slate that waits for experience to fill it. This concept was meant as a direct challenge to his mentor, Plato, who wrote that memory and experience were already present in the mind at birth and that learning was actually a form of remembering, that is, contacting the ideal realm of the Forms, where everything is complete. This processes was dubbed Anamnesis, and as we shall argue a form of this processes indeed occurs as brain development interacts with exposure to the environment. To press the metaphor, the brain may be a blank slate, but it is still a form of slate, complete with all the limitations and advantages of being slate.

Ideas on the material nature of learning and memory remained dormant for the next two thousand years, and though volumes of philosophical writings about the nature of the mind and its place in the cosmos were produced all over the world, it was not until the advent of better technology enabling the observation of new aspects of nature and the formulation of new kinds of questions. Advances in science and mechanics which allowed precise measurements of time, distance, and motion allowed psychologists to build apparatuses for measuring mental events and led to the advent of scientific psychology when Wilhelm Wundt established the Institutfür Experimentelle Psychologie in 1879 at the University of Leipzig. Here the focus was to measure the structure of cognition and emotions that were a reflection of the mind by using quantitative and reliable methods. Wundt regarded his school of thought ‘Voluntarismus’ or “Lehre von der Bedeutung des Willens” translated as “science of the relevance of the will” (as opposed to Vernunft/reason); the process of organizing the mind.

Edward Titchener, a student of Wundt, was particularly energetic, bringing to the United States this theoretical system which he renamed ‘Structuralism’ . According to Titchener the task of psychology was to identify the basic elements of consciousness in much the same way that physicists break down the basic particles of matter. For example, Titchener identified four elements in the sensation of taste: sweet, sour, salty, and bitter. The principal tool in these investigations of the physiology of sensation and perception of the (human) mind was introspection.

This did not sit well with William James [61] who was more interested in the function of consciousness than its structure. James insisted that the only means available for such study was to observe and objectively measure behavior and its consequences; any attempts to interpret the animal ‘mind’ therefore was anthropomorphisms. Further, James emphasized that behavior was a process and that even discrete acts were not static, but both cumulative and emergent in nature.

Psychologists traditionally have approached the question of individual variation very differently from behavioral biologists. The former are interested in mechanism and development while the latter in function and evolution. To psychologists the concept of life stages has always been central to the study of behavior and only recently is being recognized as important by behavioral biologists. This is curious as the concept of life history stage was central to the concept of imprinting as developed by Konrad Lorenz [62] The idea that life histories are divided into a series of sensitive periods during which specific stimuli have formative roles is a basic psychological principle. However, behavioral ecologists typically have focused on adult individuals, in other words those rare individuals that manage to survive to a reproductive age. The importance of this observation cannot be overemphasized, for the elements that lead to the survival of these few individuals has not been a subject of investigation among behavioral ecologists. One of the few exceptions to this has been the study of bird song [63].

Variability has to be unpacked and carefully sorted. For example, if we restrict ourselves to the single issue of stimulus-response, there are two questions, both of which have been investigated extensively by psychologists at the level of the individual: differences in stimuli leading to a constant response versus constant stimuli yielding different responses. When we go beyond individuals to groups of individuals (whether inbred or not) we find that manipulated groups (or strains) may show the same average phenotype for each level of the environment with differences among environmental levels. Alternatively, we may find that different levels of the environment show the same average phenotype with differences among genotypes. Thus, with very few exceptions, results of analyses such as the ANOVA are of little use when trying to understand natural systems (vs. artificial systems). Most effort has been expended on the genetic bases of behaviors, creating a vast literature that, in time, will show little import.

The G X E paradigm and its modern variants, such as genome-wide association studies, quantitative trait nucleotides, and whole-genome sequencing holds that phenotypes, including behaviors, must have a genetic basis. However, extensive and expensive research has now established that the idea that specific genes determine traits is more proof by conviction than proof by substance. There is no question there are some disease phenotypes are due to single genes, but all are rare; perhaps the best example is the parent-of-origin complementary Prader-Willi and Angelman syndromes. Further, ‘back’ mutations are even more rare [64]. We now know that multiple genes are involved in nearly every trait/disease. We also know now that, in most situations, it is how the genome is regulated, not the nucleotide sequence itself that is important. This means that the environment (both the internal milieu and the external environment) must be considered.

The heterogeneity inherent in both organisms and their environments across the life cycle defy simplistic analyses. This is the crux of plasticity and has been the object of much thought and study, particularly within psychology. This is particularly true when considering how plasticity might be related to the physical nature of learning and memory.

The Scottish philosopher Alexander Bain was the first person to propose that learning and memory must not only be associated with physical changes to the structure of the brain, but that they may actually exist as alterations or “specific growths” in the junctions between cells [65]. James, twenty years after Bain, wrote in his seminal work, *The Principles of Psychology*“…the phenomena of habit in living beings are due to the plasticity of the organic materials of which their bodies are composed” [61]. In the same work, James would go on to propose a law of association, one anticipating the major work of Hebb a half-century later: “When two elementary brain-processes have been active together or in immediate succession, one of them, on reoccurring, tends to propagate its excitement into the other” (Volume 1, p. 105). Such an associational structure would lead other astute investigators to conclusion about the physical arrangement that must exist to support this kind of associativity.

A practicing neurologist before going on to develop psychoanalysis, Sigmund Freud had begun in his notebooks an endeavor he called his “project for a scientific psychology” [66]. Here he speculated about how the structure of the nervous system could be arranged to support changes at an anatomical level that must surely exist to support changes observed at a behavioral level after learning had altered responses to specific stimuli. Indeed, he sketched a small circuit of six neurons connected by what are obviously synapses, junctions that appose but do not come into contact. He indicated the direction of information flow through this circuit and speculated about how changes in these junctions could change how stimuli and reflexes propagated through the system. Though Freud and James did not meet until 1909, a year before James's death [67], Freud was familiar with James's work, mentioning his and Danish physiologist C. Lange's theories of emotion from 1887 [68].

Further developments in the concept of plasticity had to again await further improvements in technology. Diagrams like those in Freud's notebooks would appear in the literature again shortly with the writings of Ramon y Cajal and Camillo Golgi. Cajal observed neural tissue using novel staining methods and powerful light microscopes unavailable earlier and produced exquisite drawings of the neural networks he had observed along with his thoughts on how information must flow from cell to cell through their cable-like processes. Donald Hebb (1949) drew on older ideas (including those of James) to express most clearly the suspected circumstances required to obtain for neurons to alter their physical connectivity in response to activity. He said, “When an axon of cell A is near enough to excite cell B and repeatedly or persistently takes part in firing it, some growth process or metabolic change takes place in one or both cells such that A's efficiency, as one of the cells firing B, is increased”(Pg 62) [69]. This form of plasticity has come to be known as Hebbian, though other forms of long-term changes exist in mammalian neurons besides modifications to synaptic weights.

Neuronal plasticity is the other great substrate for differential survival via natural selection. In genetics, the idea espoused by Weismann that the germline is insulated from the environment laid to rest for a hundred years the earlier notion, most famously associated with Lamarck, that information acquired by the adult animal can be hereditarily passed on to offspring which can then develop and mature with the novel trait expressed [70]. Following customary usage, in what follows we will associate the inheritability of acquired traits with Lamarck even though we acknowledge that the view was widespread in the nineteenth century and subscribed to by many others including Darwin [71] and what was truly original with Lamarck was the view that there was a positive correlation between the occurrence of a variation and the fitness change it induced [72].

Indeed, the idea that stable units of heredity exist in the brain has been expressed most clearly in the late 19^th^ and early 20^th^ century [44,73]. The modern idea of the ‘meme’ [74], or unit of mental information that can be transferred from organism to organism in an analogous way to the transmission of genes, expresses the vertical and horizontal nature of information transmission from brain to brain, even from unrelated individuals. We now know that environmental factors can physically alter gene transcription and translation, either by the methylation of cytosine molecules or the acetylation of the histone proteins that coil and organize chromatin. Such alterations to the genetic material may not change how that information is expressed, how it interacts with the environment, or how that genotype's phenotype comes to be expressed.

Thus, plasticity of the brain is preeminently Lamarckian in character – skills and knowledge acquired by an adult can be taught to children through education. This is indeed the transmission of acquired information and forms the basis of the Baldwin effect [73] whereby “Characters individually acquired by members of a group of organisms may eventually, under the influence of selection, be re-enforced or replaced by similar hereditary characters” [75]. Neural plasticity is thought to be involved in the acquisition and expression of all behaviors, including so-called instinctual ones, during the lifetime of an organism. As Hubel and Wiesel demonstrated in the cat, brain regions involved in early-stage sensory processing require environmental input to organize themselves appropriately [76]. This is a form of pre-programmed neural plasticity that continues after primary development. The organization the nervous system is such that without the expected input during the critical period, proper wiring cannot be established.

All animals, including humans, must have the capacity to acquire information quickly and store it for long periods of time. Animal signals, like language in humans, are a highly compressed form of information transmission. This requires synaptic plasticity that, along with natural selection, is subject to built-in mechanisms for mutation or variation. In this manner, for example, the information content moving from one brain to another is constant yet the fine structure of the receiving organ is different from the sender so the array of synaptic modifications will arrive with new associations and contexts, preserving as well as altering that unitary piece of information [77,78]. Since these arrays of synaptic modification are stable and can overlap across generations, the living brain matter is the source of phenotypic variation held by the information stored in neural plasticity. Such considerations are well represented in the developmentalist tradition [79-83], ideas that would benefit biologists seeking to understand how development, phenotypic variation, and inheritance interact.

### Potholes versus Sinkholes

There are many examples of missed and/or forgotten messages that have had to be re-discovered. We present just two examples, one from biology and the other from psychology. Woltereck's concept of Norms of Reaction (sometimes mistakenly referred to as the Reaction Norm) in the early 1900's lay fallow until the work of Schlichting and others in the 1980s in spite of its advocacy by Dobzhansky in the 1950s as noted earlier [16] with Lewontin being a notable exception [84]. John Garcia in the 1950's demonstrated long latencies between stimulus and response in conditioned taste aversion. Because his results did not follow the basic principles of classical conditioning of the time, they were ignored for several decades.

Such potholes, particularly in the interdisciplinary behavioral sciences, represent a lack of appreciation of the heritage of the constituent fields. For example, in the past decade there has been a dramatic increase in imbuing animals with human traits. Such anthropomorphism is not new. Indeed, the last time this battle was fought (and won) was as structural psychology (Wundt and Titchenor) gave way to functional psychology (James, Baldwin, and Lloyd Morgan) a century ago. In the present context it is particularly interesting that the source of re-emergence is found principally in the work of evolutionary biologists and behavioral ecologists. Ethology arose in Europe, with its primary questions of evolution and survival value. Then (and now) leads one to question whether the many contributions of North American psychologists and animal behaviorists whose work focused on causation and ontogeny of behavior are appreciated. In a real sense, the suggested synthesis of Tinbergen has never really taken root [85].

Of greater concern are recent research endeavors that, in the authors’ opinion, are sinkholes. When anthropomorphisms re-direct research into areas of doubtful heuristic value, they damage the field. The surge in recent papers attesting that, because individual animals vary, they have a ‘personality’ just as much as do humans, is an example. In this instance the use of statistics to demonstrate individual variance is neither logical nor novel and assertions that even insect larvae and crabs possess personality lead us nowhere [86]. The fact that individuals vary has long been known, being a pivotal point in Darwin's selection theories. It has been reaffirmed in all living organisms [87], even in isogenic animals [88,89], including parthenogenetic species [90-93] or inbred, domesticated species. It is our firm opinion, based on a survey of the evidence, that animals do not have personalities in the strict definition of the word. Individual differences and diversity does not amount to valid attributions of personality. Rare exceptions may occur, but as a generality, we are skeptical the field of animal personality will lead to insights into the mechanisms and development of behavior [94]. Of particular concern is the logical extension of personality to animals. Personality assumes personhood, a quality that does not presently encompass non-human animals. A sure consequence is the likelihood that, given the vigor and persistence of animal rights advocates, relaxation of semantics will lead to an increase in regulations and eventual demise of the study of animal behavior.

### Epigenetics

It is important to note that epigenetics is a perspective, not a set of techniques. There is a tendency to think that the study of epigenetic mechanisms can only be conducted at the level of the gene. This is an overly narrow definition and ignores much of the history detailed above. Indeed, epigenetics encompasses both the mechanisms at the molecular level as well as the outcomes at the level of both the individual organism and the evolution of the population. Some terms become useful before proceeding. It is also useful to consider that inherent in epigenetic analyses is the developmental concept of genotype-environment interaction [4,5] or G X E_D_[11] rather than the biometric approach (G X E_B_) of Fisher; the elemental difference is contained in a quote from a letter of Hogben to Fisher: “What I am worried about is a more intimate sense in which differences of genetic constitution are related to the external environment in the process of development.” (Pg. 739) [11].

**A. Nomenclature.** One of the principle problems with the (re)discovery of research topics is the words people use. Semantic problems figure strongly in the nature-nurture debate [9,95]. The same can be said for epigenetics. Here then are a few definitions that we have found useful when discussing epigenetics.

*Molecular Epigenetics*. Prior to the 1930's, the gene as the unit of heritable material was a theoretical concept without a physical identity. This did not mean, however, that the environment shaped the phenotype. The ancient idea of epigenesis (from the Greek) had been supplanted by the 18^th^ century with preformationism or the belief that organisms existed completely formed in the egg and sperm. Indeed, Wolff's *‘Theoria Generationis’ * in 1759 [1,2]) observation that organisms develop in stages, from undifferentiated to differentiated complexity, in a gradual and emergent process, laid the groundwork that the organism-environment interaction might redirect these developmental trajectories. Lamarck codified this in 1809, which even Darwin came to accept, beginning with the Fifth Edition of the *Origin*[96].

The theory of the germ plasm and the postulation of heritable units led Weismann [14,97] to reject all inheritance of acquired characters; however, his famous experiment of cutting off tails of 68 mice failing to produce a tail-less mouse was a flawed and incorrect test of the hypothesis. We now know that Weismann posed the wrong questions. Like *natural* selection, the mechanism by which Darwin's theory was based, *artificial* selection had been used for centuries to select for traits that were perceived as beneficial, developing a host of strains of cattle, goats, pigs, horses, plants etc. Thus, for the lack of a mechanism Lamarck failed. What is exciting is that we now know that, in an important sense, the Lamarckian view was correct and complements the conventional view of heredity, it was simply promoted too early and his ‘mechanism’ has only recently been discovered (molecular epigenetics).

Another common misconception is that Conrad H. Waddington coined the term ‘epigenetics’ [98], with some going so far as to exalt him as the ‘father’ of the field of epigenetics. This is not so. It is possible to trace the term ‘epigenetics’ *sensu strictu* in its modern use to the late 1800's. The following quotes from Wheeler [1] attest to this fact: “During the closing half of the eighteenth century it became clear to thinking men that individual organisms always have an epigenetic origin from preexisting individuals (Pp. 279)…and…“He who finds little difficulty in passing from the simple to the complex, from the homogeneous to the heterogeneous, will take an epigenetic view of development.”(p. 282).

Waddington's fundamental contribution was to propose the term “epigenotype” as a concept of how genes might interact with their environment and give rise to the phenotype [98]. It is in this sense that the term epigenetics is commonly used in molecular and developmental genetics today, namely, “the study of the mechanisms of temporal and spatial control of gene activity during the development of complex organisms” [99].

*Molar Epigenetics*. The term ‘molar’ is taken from William James, who used it to connote the emergent properties of developmental processes, and contrasted this with the view that properties and processes can be reduced their underlying components. This essentially is the preformation vs. epigenesis debate beginning before (see above) Plato and his student Aristotle [1]. The first arose from early evolutionists who asked how, within a species, different environments could shape different phenotypes. This area of study fell out of favor for about 60-70 years in European and American science. Interestingly, it continued as a major field of study in Russia and was represented in small part in this country in the work of Theodosius Dobzhansky and his students, most notably Richard C. Lewontin [100]. It was also a major research endeavor in the United States, being personified by Gilbert Gottleib [101,102], Zing Yang Kuo [103], Daniel S. Lehrman [95,104,105], Jay S. Rosenblatt [106-109], and Theodore C. Schneirla [110-112]; for a general introduction to this body work, see [113]. Today, it has re-emerged as a vigorous area of research among evolutionary biologists and behavioral ecologists. New research on the origins of polymorphisms and polyphenisms has led to a concept now commonly referred to as ‘phenotypic plasticity’, which is considered one of the driving forces in the relatively new union of developmental biologists with evolutionary biologists (‘Evo-Devo’) [114,115].

Waddington [116] recognized that “The greater part of biospsychological development takes place during periods of the life history much later than the embryonic.” (p. 16). In this regard it becomes helpful to classify the types of epigenetic modifications. There are two basic categories [117].

Context-Dependent Epigenetic Modification (E_c_). Consequence of exposure usually occur early in life or during adolescence, but may result from trauma at anytime in life. Common examples would be exposure to endocrine disrupting chemicals *in utero* or smoking during childhood and adolescence. In the first instance the onset of disease manifests later during the individual's lifetime while in the latter instance, the deleterious effects of smoking decline with time *only if the individual is no longer exposed to the stimulus*. This type of epigenetic modification can be perpetuated across generations by simple persistence of the causal environmental factor such that each generation is exposed to the same conditions. However, if the agent/exposure is ameliorated it will not be transmitted to the next generation.

Germline-Dependent Epigenetic Modification (E_g_). Germline-dependent epigenetic modifications are fundamentally different than Context-dependent epigenetic modification in that the epigenetic imprint has become independent of the original causative agent. That is, the epigenetic modification is transferred to subsequent generations because the change in the epigenome has been incorporated into the germline. Thus, the effect is manifested each generation, even in the absence of the causative agent. In such instances the DNA methylation of heritable epialleles (a group of otherwise identical genes that differ in the extent of methylation) are passed through to subsequent generations rather than being erased as occurs normally during gametogenesis and shortly after fertilization.

The defining distinction between Context- and Germline-dependent epigenetic modifications lies in the timing and persistence of the exposure. Exposure to environmental or psychological stressors will bring about change in the epigenome, but the transmission of the effects of that exposure can occur in two basic ways. Context-Dependent epigenetic modifications are in direct response to the stimulus. Thus, an endocrine disruptor in the environment will induce changes in all individuals that are exposed to it and, as long as the environment stays contaminated, further generations will also exhibit the modification. On the other hand, Germline-dependent epigenetic modifications can be transmitted to future generations without the requirement of additional exposure. In such instances removal of the contaminant will not result in resumption of the original, non-modified state because the modification has become part of the germline and will pass to all future generations. Thus, only Germline-Dependent epigenetic modifications are truly transgenerational in nature.

**B. The Study of Interactions.** To study interactions it is best to first agree on what is a phenotype. For our purposes a phenotype consists of multiple traits; each trait is defined as any measurable aspect of the individual. In general, our understanding of a particular phenotype increases proportionally with the number of traits that are measured in the same individual. Selection of the particular morphological, physiological, behavioral, and brain nucleus traits should be predicated on the literature and demonstrated to be important to the question at hand. The same principle applies to genes in that individual genes only have meaning in the context of other genes within and outside their functional categories.

When multiple traits of complex phenotypes are examined as a unit (e.g., the suite of genes known to be involved in sex determination and gonadal differentiation or the neural circuitry underlying sociosexual behavior), conventional analytic and presentation methods make it difficult to quantify and illustrate the information. Scarpino et al. [118] introduced an adaptation of established methods for analyzing complex data sets that takes a computational systems biology approach, integrating data, analysis and visualization. Our method of depicting complex phenotype analysis, which we have called the Functional Landscape Method, can be viewed as a recent addition to the long history of imagery to depict complex concepts in all areas of science. Well-known images in Biology would include Waddington's developmental landscape depicting the genes that shape tissues and, more recently, Nijhout's schematic of the importance of context in trait development [119,120]. Similarly, in psychology, there is Gottesman's depiction of the contribution of genes to cognitive ability and that of Grossman et al. illustrating how genetic and experiential factors push the individual to thresholds of pathology [121,122]. Notably, all share the use of three dimensions to illustrate complex traits whose individual components are two-dimensional in nature. The shared quality of these images is predicated on the fact that the mind can process 3D comparisons much better than complex bar graphs or tabulated results, a fact verified many times in cognitive psychology.

The Functional Landscape method enables one to visualize the composite phenotype for each group, and thus compare the phenotype between groups. The peak of each node in the landscape is calculated as the percent of maximum from the highest group mean. The width of each node was adjusted to optimally fit the number of nodes in each landscape and has no statistical significance. A percent change landscape is then created to visualize the differences between groups. The direction of the node, either below or above the plane, indicates in which direction the mean is influenced by the effect of treatment or group. A node above the plane indicates the mean in the treated group is higher than the mean of the control group and vice versa. This method allows one to visualize the composite change in the phenotype of a control group to that of a treated group. Functional landscape analysis can also be implemented to display the most influential phenotypesin each category (e.g. brain, behavior, physiology) (c.f., Figure 2D in [123]. This ‘Essential Phenotype’ is calculated on the entire dataset, first utilizing discriminate function analysis to determine the measures that best separate each group from one another. The three most influential measures from each category are then selected for visualization of Essential Phenotype to depict the measures that are most influential in determining group differences.

**Figure 1:**
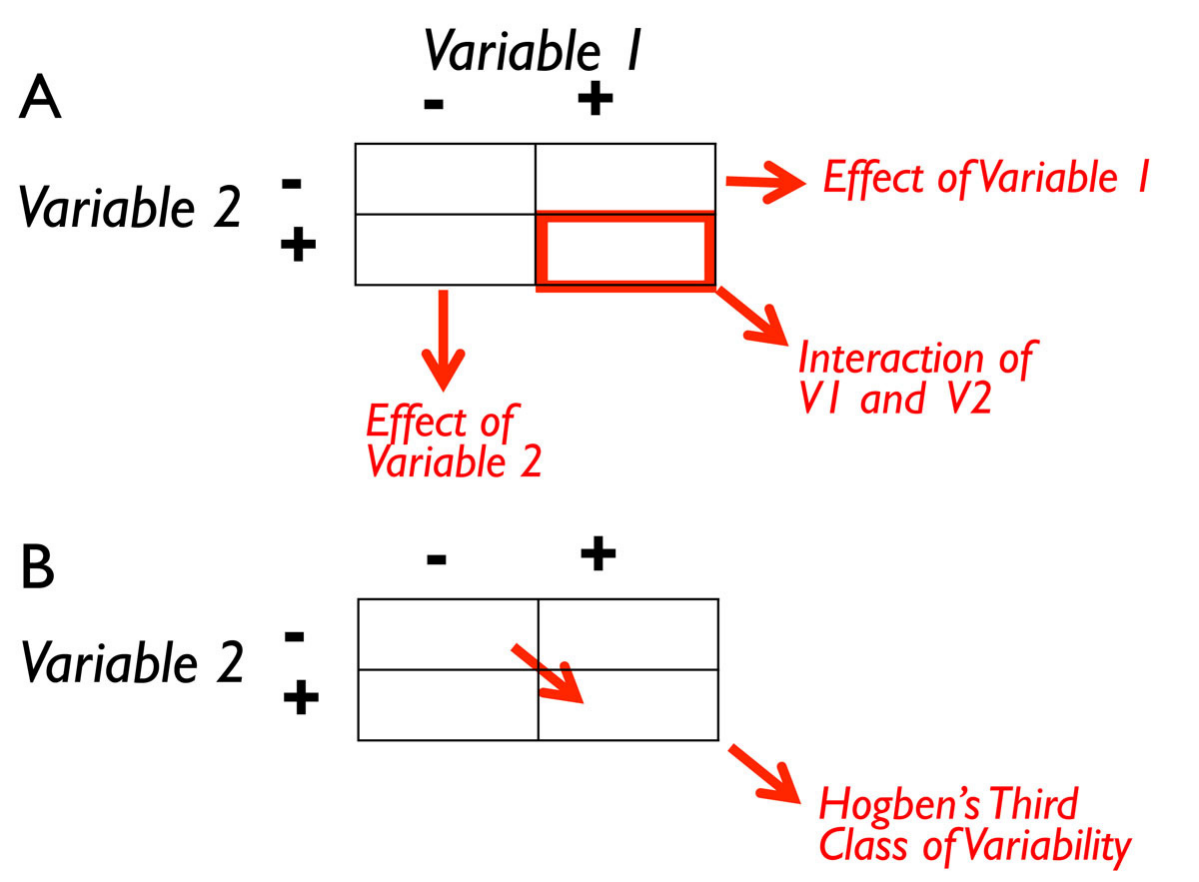
Contrasting analyses of biological phenomena. (A) Fisher developed a mathematical approach to analyze the distribution of traits (analysis of variance). This deconstructed phenotypic variability using a linear model into two main effects (Variables 1 and 2) and their interaction with the goal of understanding the relative contributions of each variable. (B) Hogben proposed that any analysis of biological variance would require consideration of genetics, environment, and development simultaneously. In particular, under certain circumstances where “the combination of a *particular* hereditary constitution with a *particular* kind of environment” (Hogben, 1932), as is the case when variables that occur generations apart, may create novel phenotypes. This principle is termed ‘synchronicity’ and focuses on the experience of two or more events that are unlikely to occur together by chance, yet when experienced together are meaningful. Thus, the combined effects transcend a simple interaction of two independent and unrelated events.

There are three classes of variability when studying interactions (**Figure** 1). The first and second can be analyzed by the usual contingency tables typical of ANOVA. In a typical 2-way ANOVA with two factors (1 and 2), the first class of variability would be due to the effects of each Factor. The second class of variability would be the interaction of the two factors. It is possible that studies may demonstrate significant effects of Factors 1 and 2 (or only of one) and significant (or insignificant) effects of the interaction of Factors 1 and 2. If we delineate the Factors as Gene and Environment, research shows that although there are monogenic diseases, these are rare and the genetic contribution to most diseases is only about 2-5%, and also that multiple genes are involved in nearly every trait/disease [124,125]. In this regard the study of interactions in the emerging field of environmental epigenetics is worth mention. Let us assume that life is subject to two types of epigenetic modifications: context-dependent epigenetic modification and germline-dependent epigenetic modifications (see above for definitions).

The *first class of variability* represents the consequences of manipulations; in the example that will follow, this is **either** the administration of the fungicide vinclozolin to pregnant females or the exposure to chronic restraint stress (CRS) during adolescence of animals 3 generations removed. This class of variation reflects the independent statistical comparison between the control condition and each of the two manipulations respectively; this is also known as the main effects in an analysis of variance statistical test. The *second class of variability* would the interaction term. In this instance the effect of CRS in animals from the vinclozolin-lineage. The *third class of variability* is that of Hogben [4,5]. Unlike Fisher, Hogben's developmental consideration of gene X environment interactions “arises from the combination of a particular hereditary constitution with a particular kind of environment” [4], (p. 98) (see also [11]). As mentioned above, Hogben's addition of development into genotype-environment interaction is designated as G X E_D_. The most appropriate term for this *class of variability* is *synchronicity* or the “the simultaneous occurrence of two meaningful but not causally connected events” [126]. This constitutes a new and different “order” of historical causation. In this instance the unmanipulated lineage, Non Stress versus the vinclozolin-lineage that received CRS during adolescence. This brings us to different types of ‘two-hit’ designs.

As discussed in Crews and Gore [127,128], real life consists of ‘two-hit’ or even ‘multi-hit’ experiences and the accumulation of these in an individual have consequences that can range from ‘normal’ function to debilitation. We will consider here the simplest of ‘two-hit’ designs. Classically, this takes the form of a single exposure followed by a second exposure after some period of time. Much of biological processes are underpinned by such cumulative interactions. For example, estrogen priming prior to the administration of progesterone to facilitate the expression of sexual behavior in adult female rats; estrogen priming followed by a second injection of estrogen to stimulate functional uterine development; prenatal secretion of gonadal steroids prenatally followed by a second increase in gonadal steroids during puberty necessary for normal reproductive function. Most studies of these types of ‘two-hits’ occur within the life history of an individual and focus on the consequences of the interaction. In this instance, the second class of variability can be *emergent* as defined by Mayr [129] “When two entities are combined at a higher level of integration, not all the properties of the new entity are necessarily a logical or predictable consequence of the properties of the components” (pg. 34). Thus, in most instances the third class of variability refers to significant sequential events within the life history of an individual (e.g., conception, birth, adolescence, sexual maturity). As such, the events or experiences are causally related because they are stages within a linear path (conception to death).

However, what is not captured in ‘emergent’ is the combined effects of important heritable and experienced phenomena that are not causally connected, particularly when generations separate the hits and, further, the hits are fundamentally different in nature. For example, in transgenerational epigenetic modifications the first hit may be experienced during pregnancy of an individual while the second hit occurs during the life history of descendant generations. An example is the ‘two-hit, 3 generations apart’ model we have used. In this instance the hits are different in nature (vinclozolin exposure, a germline-dependent epigenetic modification versus exposure during adolescence to restraint stress, a context-dependent epigenetic modification) and occur in different generations. In this instance there is no causal connection between the exposures (e.g., an ancestral hit may be exposure to a EDC, while the hit to the descendant may be a stressful experience). It is important to understand that the nature of the hits must be different; that is, exposures to different EDCs that operate by different mechanisms of action do not necessarily share causality even though they operate as EDCs. By studying combined ancestral and acquired epigenetic modifications we have another perspective on the hoary concept of nature vs nurture [9,10]. While this concept has been around for a long time, and many scientists have railed against it, it does capture the essence of a situation where important heritable and experienced phenomena that *are not causally connected* co-occur. What matters is the history of developmental processes, in our examples operating sometimes transgenerationally. When this happens the resulting altered phenotype cannot be attributed to either the heritable component or the experienced component. In this instance the impact of epigenetically induced transgenerational history changes how descendants respond to events in their own life, particularly how they perceive challenges. Just as environment cannot be reduced to a single factor, inherited traits cannot be reduced to gene(s) and epigenetics without consideration of its intergenerational history.

### Five examples: from simple to complex

There is a voluminous literature on the development of behavior in animals that goes back centuries. From the earliest writings the role of experience was noted. However, it was not until the mid-1900's that scientists began to appreciate that life was punctuated by particularly sensitive (nee critical) periods in which certain experiences have considerable impact on subsequent development. Inherent in this perspective was the understanding of complex progression and that each moment in time is based on what has gone before and, at the same time, sets the stage for what will follow. Presented here are five instances focusing on the development of the phenotype.

**A. Incubation temperature during embryogenesis in the turtle results in two distinct phenotypes, male and female.** The enormous diversity in sex-determining mechanisms has revealed that a constellation of evolutionarily conserved genes orchestrate whether testes or ovaries will be formed from the genital ridge [130,131]. Whatever the switch or trigger (e.g. *Sry* in humans), this in turn engages a primary gene “cassette” of evolutionarily conserved, functionally related genes that interact to determine gonadal fate [131]. The nature of these interactions change through development and this cassette engages other cassettes of integrated gene assemblies, such as those responsible for sexual differentiation of secondary and accessory sex structures. Thus, the developmental decision of male versus female does not flow through a single gene but is instead determined by a parliamentary system involving networks of genes that have simultaneous inputs to several components of the downstream cascade. Systems with different degrees of the inherited and environmental influences could all operate this way, merely by varying the inputs to the networks.

Temperature-dependent sex determination (TSD) is a prime example of phenotypic plasticity in that the temperature of the incubating egg determines the nature of the gonad. In the red-eared slider turtle (*Trachemys scripta*), gonadal sex is determined by the incubation temperature of the eggs during the mid-trimester of development, known as the temperature-sensitive period (TSP). Eggs incubated at 26 °C become males (= male-producing temperature or MPT) while eggs incubated at 31 °C become females (= female-producing temperature or FPT). At the intermediate temperature of 29.2 °C (= threshold temperature) a 50:50 sex ratio is produced (Wibbels et al. 1991) [132]. It is important to note that the range of temperature between all-male and all-female clutches is less than 1 °C (Crews et al., 1994).

Precisely how the physical signal of temperature is transduced into a biological signal that ultimately results in sex determination remains unknown. Several genes (or gene networks) involved in mammalian sex determination system also exhibit gonad-typical expression patterns in response to temperature in the red-eared slider. At the onset of the TSP these genes begin to exhibit differential expression patterns between FPT and MPT, suggesting that the transcription of these genes are critical in sex determination and closely regulated by temperature. Remarkably, if taken during the TSP, embryonic gonads cultured *in vitro* are also capable of responding to surrounding temperature with sex-typical gene expression and appropriate gonadal differentiation, suggesting the capability of isolated embryonic gonads to sense surrounding temperature and regulate gene expression [133,134].

**B. Early and late experiences contribute to the adult behavioral phenotype.** As in the red-eared slider turtle, incubation temperature in the leopard gecko (*Eublepharis macularius*) determines gonadal sex. The pattern is different however. Low (26 °C) and high (34 °C) incubation temperatures produce only females while intermediate incubation temperatures produce different sex ratios; 30 °C produces a female-biased sex ratio (25:75 or Tf), and 32.5 °C produces a male-biased sex ratio (75:25 or Tm).

Incubation temperature not only establishes the gonadal sex of the individual, but also accounts for much of the within-sex variation observed in the morphology, growth, endocrine physiology, and aggressive and sexual behavior of the adult (reviewed in [135]). For example, males in general grow more rapidly and are larger than females from the same incubation temperature; Tm males however grow more rapidly and to a larger size than do Tf males. Hatchling, young, and adult Tm and Tf males do not differ in circulating concentrations of androgens. Estrogen levels do differ significantly, however, with Tf males having higher levels than do Tm males. Despite this similarity in circulating androgen levels in adulthood, males from the two temperature morphs differ significantly in their scent-marking response to exogenous hormones in adulthood, indicating neuroendocrine differences between the Tf and Tm males. There are also between-sex as well as within-sex differences in glucocorticoid levels in response to stress. Females have higher circulating levels of corticosterone than males, but, for both females and males, Tm individuals have significantly lower levels than do Tf individuals. Brain neurochemistry is also influenced by incubation temperature. For example, a significantly higher number of tyrosine hydroxylase immunoreactive cells are found in the ventral tegmental area of sexually inexperienced Tf versus Tm males that had been castrated and androgen-implanted, suggesting that embryonic temperature plays a role in differentially organizing dopaminergic systems of the temperature morphs. This is supported by the finding of significantly higher dopamine levels in the nucleus accumbens of Tf males compared to Tm males that have interacted with a receptive female across a barrier. Finally, sexually experienced Tf and Tm males both show strong preferences in a Y-maze apparatus to females or their odors, but the type of female they choose depends upon their incubation history. For example, given the simultaneous choice between two females from different incubation temperatures, Tf males prefer high temperature females (34 °C), while Tm males prefer the low temperature or Tf females. Among females, Tm females are less attractive to males than are Tf females and will even attack males, a typically male pattern of aggression.

**C. Distinguishing the Contributions of Prenatal and Postnatal Factors to Adult Male Sexual Behavior in the Rat.** Most animal studies focus on manipulations in particular life stages (= sensitive periods) and assess their consequences on later stages. Each of these periods is influenced by the context in which the individual finds itself. Prenatal development is classically considered a period in which the body and brain are organized by hormones. In the prenatal period the uterine environment includes the physiology of the mother and fetal neighbors, a phenomenon known in the literature as the intrauterine position (IUP) effect (assuming multiple young in the pregnancy). The environment of the mother (e.g., ingested products, exposures to stressful social conditions and toxicants) will affect embryonic development, but the extent and nature of their impact will depend upon the developmental stage of the fetus(s) at the time.

The IUP effect posits that secretions of fetal neighbors (in particular testosterone from the male fetus) can affect both physiology and behavior of the offspring when they are adult (Ryan and Vandenbergh, 2002) [136]. This effect has been observed in humans (dizygotic twins) and in agricultural and wild animals, but the bulk of research has been conducted on lab mice and rats (c.f., [137-140]). Thus, a female fetus located between two males (a 2M female) is exposed to higher levels of androgen produced by the neighboring males compared to a female fetus located between two females (a 2F female). As adults these 2M females have lower estrogen and higher testosterone levels, have a masculinized phenotype, are less attractive to males and more aggressive to females, and produce litters with significantly greater male-biased sex ratios relative to 2F females. However, it is also the case that this literature does not take into account the postnatal sex ratio of the litter. This is important because the prenatal sex ratio of the litter influences circulating levels of testosterone in the pregnant female [141,142], and that the postnatal sex ratio of the litter determines the amount of maternal licking of offspring [143]. Thus, it is necessary to establish how the prenatal and postnatal developmental periods, and the environmental factors in each period, differ and interact. The effect of progressive developmental changes on behavioral outcomes has been studied in precocial birds, in which the difference in timing of developmental periods is a major factor in differentiating filial imprinting versus sexual imprinting [144]. By manipulating the postnatal sex ratio of the litter and controlling for the prenatal sex ratio, it is possible to assess the relative influence of early prenatal experiences *in utero* (indicated by pup sex ratio at birth), versus postnatal experiences due to litter sex ratio, using male sexuality in adulthood as the endpoint [145]. Specifically, by noting the sex ratio at birth (reflecting the intrauterine sex ratio), culling and reconstituting litters on the day of birth, and assigning them to mothers other than their natural mothers, it is possible to deconstruct the progression of developmental experiences. The results show that the litter composition (the number of male and female littermates) is more important than by the prenatal litter ratio in the sexual activity of males when they were adults. Further, we found males reared in a female-biased litter are less attractive to females. This appears to be compensated for in that these same males were more efficient maters that males raised in male-biased litters.

What is it about the litter that may lead to these differences later in life? If it is not IUP or maternal care, what is it? Jeffrey Alberts and colleagues have studied the dynamic movements of pups in the litter and how it changes through development. The huddle is characterized by seemingly endless efforts of individual pups trying to get to the center, where it is warmest. This constant flux has a pattern however, and a recent study has yielded a surprise. In infant rats and mice pockets of brown adipose tissue (BAT) in the back are thermogenic and provide targets for nest mates seeking warmth, increasing the cohesiveness of huddling groups [146,147]. There are sex differences in BAT regulation and thermogenesis, with female mice being slightly warmer than are male mice. Creating temporary artificial litters of varying numbers of males and females and mapping the movements of individuals in the huddle, it was discovered that the females’ higher temperatures make them more attractive huddling targets. This, in turn, resulted in the sexes assorting themselves differently in the huddle, with females huddling more with females and males with males. This sex difference in thermogenic behavior likely reinforces other sex differences in earlier sensitive periods (prenatal).

While there is no doubt that experiences early in life lay the foundation for an individual's behavior as an adult, exactly how this happens continues to be a mystery. Very few experiments to date have attempted to deconstruct the life cycle in a manner that isolate how particular experiences during a single life stage affect development *independent* of surrounding life stages. That is, the relative influence of the specific sensitive periods and associated stimuli are rarely dissociated from one another. Controlling for the cumulative nature of multiple factors during development is a challenge, requiring a well-established animal model that is both abundant and can be experimentally manipulated to distinguish between the component elements. When these normally seamless events are assessed separately, without the confound of the other, the importance of the composition of the litter (= family) in shaping adult behavior is evident.

**D. Distinguishing the Contributions of Specific Genes and Litter Composition on Brain and Behavior in the Mouse.** It is also possible to deconstruct the relative contributions of the litter and of specific genes by using knockout mice that lack functional copies of a gene(s). In many such strains, the knockout (KO) individual is generated by breeding heterozygous animals, usually yielding litters having a Mendelian ratio of 1 homozygous, 2 heterozygous, and 1 null or knockout. Such animals are used extensively in gene-brain-behavior research. What is usually not considered, however, is the sex ratio or the genotype ratio of the litter. We have conducted such a study using genetically modified mice in which functional estrogen receptor α (ER) is lacking, also known as the ERKO mouse [148]. After sexing (male vs female) and genotyping (by PCR) the offspring within the first two days of life, litters were reconstituted to control for both sex ratio (creating litters that were all-male, all-female, or equal numbers of males and females) and genotype ratio (all wild type pups, all knockout, or equal numbers of wild type and ERKO pups).

The consequence of litter composition during rearing on adult social and anxiety-related behaviors depends upon the whether or not the animal is raised with brothers or sisters and, in particular, their genotype. When raised in litters having either male or female ERKO littermates, WT males are more aggressive than when raised in litters containing only WT males or mixed-sex, same genotype siblings. Anxiety-like behaviors, as reflected in behavior in the Light:Dark box, is modified in WT males if they had WT sisters as litter mates, an effect absent if the sisters are ERKO females. Among females, the social behavioral profile of ERKO females is significantly different depending upon the genotype of their sisters. In both males and females the sex and genotype of the siblings in the litter affects patterns of metabolic activity in specific brain areas later in adulthood. For example, males raised with WT brothers have significantly lower mean cytochrome oxidase (CO) activity in the limbic nuclei compared to males reared with KO brothers. In females these effects were more restricted. Considering the activity of functionally integrated networks of brain nuclei, rather than brain nuclei individually, it is apparent that the pattern of metabolic activity varies depending upon the genotype of their brothers and sisters in the litter. Principal Components Analysis reveals that in both WT and ERKO males there are two principal but overlapping networks, one rostral to the other. A single nucleus, the ventromedial hypothalamic nucleus, is common to both networks, suggesting its pivotal nature in the organization of these two neural networks. Thus, both the sex and genotype ratios of the litter significantly affect the adult behavior, metabolic activity in specific brain nuclei, and the functional connectivity in functional networks of brain nuclei in genetically modified and WT mice. What is particularly interesting is that ERKO females are more similar to WT males than they are to WT mice in these aspects, suggesting that ERKO females play a male-type role in the pre-weaning sibling environment. Thus, it is the context (litter or family) in which the individual is nurtured that shapes the brain and behavior when it is an adult.

**E. Inherited and acquired experiences combine to create new phenotypes.** Although most work has focused on early life stages (prenatal and infancy), in the past decade animal research has begun exploring adolescence as a sensitive period. Adrenal activation begins after weaning and includes a drenarche or the increase in activity of the adrenal glands just before the onset of puberty. Pubarche is initiated toward the completion of adrenarche. Adrenarche and pubarche constitute adolescence and it is during adolescence that adrenal and gonadal hormones reshape the body and brain. It is as an adolescent that the individual graduates from dependence to independence, assuming the properties of maturity. The question we pose here is how events experienced during an individual's life might interact with events encountered their ancestors.

It now evident that environmental contamination has become a clear and present danger to all life processes [127,149]. Vinclozolin is a common use fungicide used extensively in agriculture and experiments have shown that it acts early in life as an anti-androgen with transgenerational properties [150,151]. How might inherited and acquired experiences combine to create new phenotypes? In this experiment, two manipulations were combined; administration of vinclozolin to pregnant female rats and, after three generations of no further EDC exposure, stressing descendant animals during adolescence. We chose chronic restraint stress (CRS) because it is exceptionally well characterized at the physiological, neuroendocrine, and behavioral levels in rats. The experimental 2 X 2 design used is often referred to a “two-hit” or “miss, miss-match”. In this manner, the four treatment groups are: [Vinclozolin Lineage plus adolescent Stress (VS), Vinclozolin Lineage with no Stress (VNS), Control Lineage with adolescent Stress (CS), and Control Lineage with no adolescent Stress (CNS)]. This design enables detection of the effect of ancestral exposure alone, the effect acquired from stress exposure during adolescence alone, and the combined effect of ancestral and acquired conditions.

**Figure 2:**
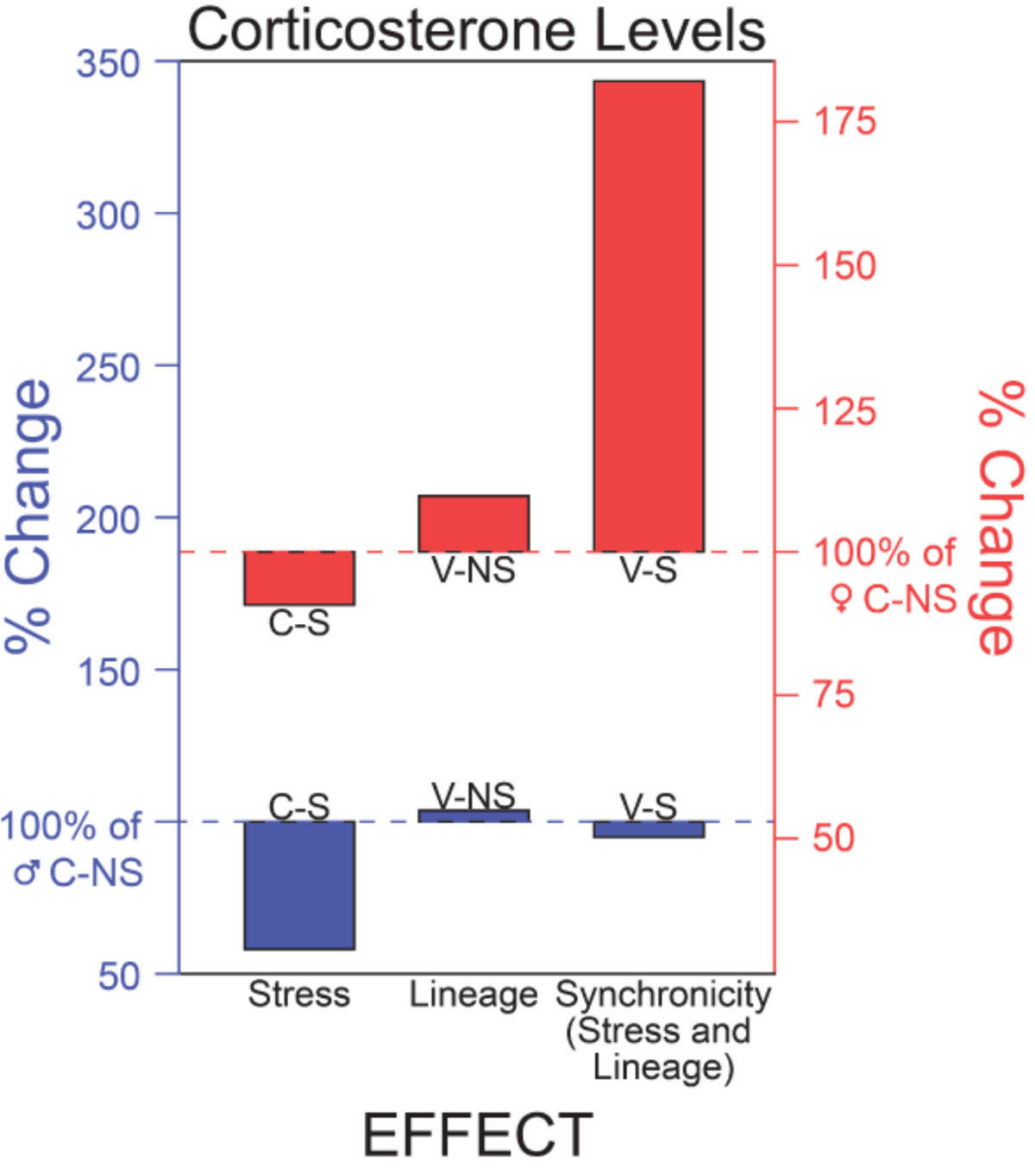
First and third classes of variability. In this experiment male and female rats were exposed in the F0 generation to the endocrine disrupting compound vinclozolin (Lineage) followed by the F3 generation exposed to chronic restraint stress (CRS) during adolescence (Stress). Circulating levels of corticosterone (CORT) were measured in adulthood. Note that the large sex differences in basal CORT levels (hatched line in females in red, hatched line in males in blue). Comparing the effects of CRS alone it becomes apparent that Stress during adolescence decreases CORT in both sexes, with males affected more than females. The effect of vinclozolin three generations previously (Lineage) has no effect. The combination of both conditions (V-S), illustrates how Lineagepotentiates the effects of Stress in females while in males, it ameliorates the effects of Stress.

There are large sex differences in a variety of phenotypic traits among control Non Stress animals [152]. This is exemplified by the sex and treatment differences in circulating levels of corticosterone (**Figure** 2). As per the literature, the baseline levels of corticosterone are different in CNS males and females, with males having significantly lower circulating levels than females. Stress during adolescence decreases corticosterone in both sexes, with males being affected more than females. The effect of ancestral exposure to vinclozolin is modest in both sexes, but the sex difference is maintained. When VS animals are compared to CNS animals, however, a striking difference is seen. That is, ancestral exposure to vinclozolin significantly increases the effects of CRS during adolescence in the descendant females, but there is no apparent effect in males. This means that when these two types of epigenetic modifications are combined, there is a profound sex difference in the scope and nature of reactivity that cannot be explained by either variable alone.

Males and females also exhibit significantly different reactivity profiles in standardized behavioral diagnostic tests, with females showing more anxiety-like behavior [152]. The only exception is the behavior of males in the elevated plus maze. Males show a stronger preference for social affiliation than do females, but both sexes prefer to associate with a stimulus animal versus an empty chamber. Only females exhibit a clear preference for the novel stimulus animal when given the choice to investigate a familiar stimulus animal or an unfamiliar stimulus animal.

There is a substantial difference in the profile of CO abundance in target nuclei, with females showing elevated activity in most nuclei. In general, control NonStress males show decreased metabolic activity in hippocampal nuclei while females exhibit increased metabolism in the medial and central amygdaloid nuclei.

Analysis of the specificity of gene expression according to sex and brain nucleus reveal a marked sex difference in the numbers of genes regulated, with CNS females showing most changes in regulation in the CA3 of the hippocampus (CA3), while in CNS males the majority of genes showing changes are in the basolateral amygdala, bed nucleus of the striaterminalis,, and CA3 of the hippocampus [152]. The greatest sex difference is found in the pattern of gene expression in the CA3 of the hippocampus of females. Interestingly, the only gene expressed in both males and females (*Mc4r*) is in this group, showing down regulation in males and up regulation in females in the ventromedial nucleus of the hypothalamus. The majority of genes affected belong to receptor class proteins and growth factors [152]. Ancestral exposure to vinclozolin up regulates the gene coding for ER (*Esr1*) regardless of CRS exposure, suggests that its’ expression may be affected by altered methylation patterns established in germline cells during embryonic vinclozolin exposure of the F0 generation. Why the massive effect of VS should be limited to the CA3 of the hippocampus in females is not known but could be due to the particular context in which the genes are expressed.

### Conclusion

It is not novel to assert that scientific progress is not linear, but in steps. It is characterized by bursts of creativity and discovery that establish the major platforms in scientific understanding at any given time. It is also not original to note that each discipline has at its core a central dogma(s) reflecting the most accepted findings. As Kuhn [153] noted, many of the ‘new’ discoveries occur when disciplines overlap, leading eventually to new dogma. Lastly, it is not unusual for some research to be outside of a zeitgeist that is not receptive to the changes such findings would impose. Thus, the **timing** of discoveries is at least as important as the discoveries themselves. Such has been the fate of ‘epigenesis’ for almost a century.

Technological advances far outstrip our ability to place discoveries in the context of larger questions. This has led to an increasing number of rediscoveries of old ideas or, what we regard as wasted energy that could have been used more wisely. For example, ‘systems biology’ today is a far cry from its origins. Indeed, ‘systems biology’ as originally developed by Paul Weiss arose from his 1928 thesis on butterfly flight and reflected the epigenetic perspective where developmental processes were the crux [154]. Today ‘systems biology’ largely consists how large data sets derived from state-of-the-art methods might relate, usually without consideration of the organisms that generated the data.

Consider as a thought experiment that there are 20,000 genes that act as discrete elements, all of which interact with one another. Consider also that there are 20,000 traits (a trait being anything that can be measured) and 20,000 environments, also discrete units. Most would suggest that such G X E or more modern algorithms would be able to determine the relative values that make up the phenotype. Yet clearly this is impossible because the real world does not operate with discrete units that are binary in nature nor is it a balance sheet. The missing element is development or more accurately, life stages. One feature of biology that makes it particularly resistant to numerical analysis such as implied above is the existence of self-reference and self-referential feedback loops employed by all biological systems. Genes can interact directly with one another, either physically on the DNA molecule through adapter proteins regulating gene expression or regulating regulation through physically modifying the chromatin, and they can also interact indirectly over multiple disparate time periods, such as when genes controlling brain development cause behavioral changes in subsequent life stages that lead to varieties of heritable or non-heritable epigenetic changes.

**Figure 3:**
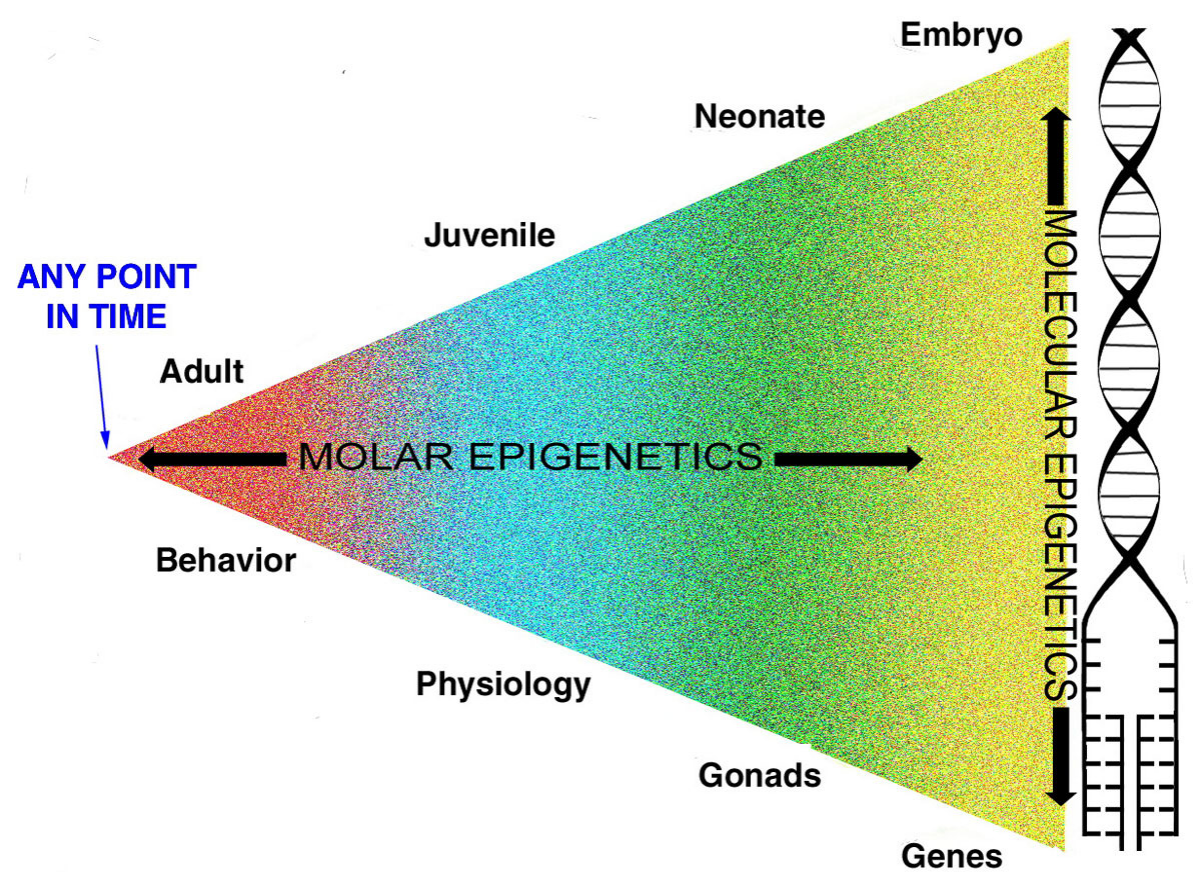
Any point in time results from those events that precede it (developmental as well as experiential). This perspective includes molecular as well as molar epigenetic events.

It is our opinion that the central question in behavioral biology today concerns the nature of development and experience. Again, this is not a new idea, but one that has been forgotten with time. As stated by Schneirla and Rosenblatt [112] social ontogeny represents *“*…the fusion of maturation (growth-contributed) and experience (stimulation-contributed) processes at different stages in behavioral ontogeny, together with the contention that the contributions both of maturation and of experience (the latter including, but not confined to, conditioning and learning), as well as the interrelations of these contributions, may differ greatly according to stage in any animal.” (Pp. 1112-3). Plasticity reflects the susceptibility as well as the capacity to change in response to internal and external cues. The external environment can be divided into physical, biotic and social elements while the internal environment can be visualized as chemical and electrical signals. By definition change only occurs after the experience (be it emergent or reactive). Therefore, plasticity is reflected in the responsiveness to experience. Life history theory is seriously lacking in its understanding of the role of experience in developmental systems. For example, most behavioral biologists tend to think of development as comprising three primary periods of sensitivity; prenatal, postnatal, and puberal. In fact, development is a series of tightly timed overlapping cascades of sensitive periods with each affecting various traits. Further, experience, once it occurs, cannot be undone or revoked; whether its’ effects erode over time is the subject for another time. All five of the examples provided make the point that while morphological development may unfold in characteristic ways, social context create stochastic inputs that will establish different trajectories that result in individuals with different physiologies, brain chemistry, and behavior (**Figure** 3).

**Figure 4:**
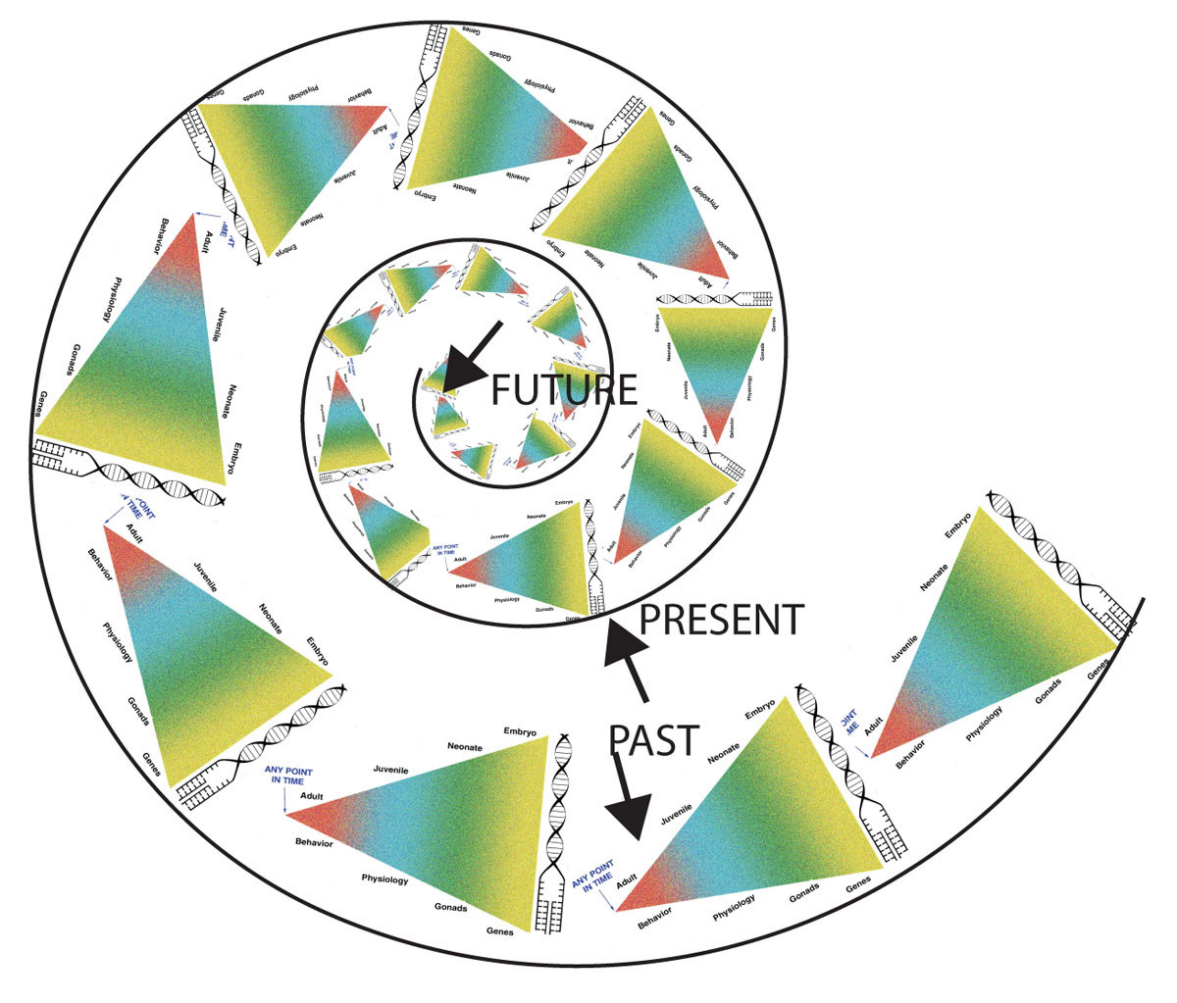
A transgenerational perspective focuses on the present, but also considers the past and projects the future. It is based on the fact that any point in time (see Figure 3) emerges from the growth and experience of the individual; this in turn, establishes the trajectory for the future. This perspective incorporates the individual's life stages, but also those of past generations.

We conclude with the following suggestions. First, all wishing to understand issues of ontogeny of behavior should know the basic literature in the relevant fields, particularly psychology and biology (e.g. [113]). To do otherwise results in risks, particularly the conduct of déjà vu studies that prove the already proven. Second, appreciate that all points in time are the result of what has come previously and set the trajectory of what will follow. It is useful to think of the entire life process as a concatenation of exposures and experiences. These experience can cross generations but are not necessarily ‘transgenerational’; hence the necessity of distinguishing between Context- vs. Germline-dependent modifications. Third, epigenetic factors can be both cause and consequence in behavioral development. Most importantly, understand that epigenetics is a perspective, and much more than molecular measures and methods. Epigenetic modifications are not singular nor are they equal. Rather, epigenetics encompasses all life processes, past, present and future [155] (**Figure** 4).

## Declarations

We acknowledge financial support for this publication by the German Science Foundation (FOR 1232) and the Open Access Publication Fund of Bielefeld and Muenster University.

## Competing interests

The authors declare no competing interests.
